# A small molecule approach to degrade RAS with EGFR repression is a potential therapy for *KRAS* mutation-driven colorectal cancer resistance to cetuximab

**DOI:** 10.1038/s12276-018-0182-2

**Published:** 2018-11-20

**Authors:** Sang-Kyu Lee, Yong-Hee Cho, Pu-Hyeon Cha, Jeong-Soo Yoon, Eun Ji Ro, Woo-Jeong Jeong, Jieun Park, Hyuntae Kim, Tae Il Kim, Do Sik Min, Gyoonhee Han, Kang-Yell Choi

**Affiliations:** 1 Translational Research Center for Protein Function Control, Yonsei University, Seoul, Korea; 2 Department of Biotechnology, College of Life Science and Biotechnology, Yonsei University, Seoul, Korea; 3 Department of Internal Medicine and Institute of Gastroenterology, College of Medicine, Yonsei University, Seoul, Korea; 4 Department of Molecular Biology, College of Natural Science, Pusan National University, Pusan, Korea

## Abstract

Drugs targeting the epidermal growth factor receptor (EGFR), such as cetuximab and panitumumab, have been prescribed for metastatic colorectal cancer (CRC), but patients harboring *KRAS* mutations are insensitive to them and do not have an alternative drug to overcome the problem. The levels of β-catenin, EGFR, and RAS, especially mutant KRAS, are increased in CRC patient tissues due to mutations of *adenomatous polyposis coli* (*APC*), which occur in 90% of human CRCs. The increases in these proteins by *APC* loss synergistically promote tumorigenesis. Therefore, we tested KYA1797K, a recently identified small molecule that degrades both β-catenin and Ras via GSK3β activation, and its capability to suppress the cetuximab resistance of *KRAS*-mutated CRC cells. KYA1797K suppressed the growth of tumor xenografts induced by CRC cells as well as tumor organoids derived from CRC patients having both *APC* and *KRAS* mutations. Lowering the levels of both β-catenin and RAS as well as EGFR via targeting the Wnt/β-catenin pathway is a therapeutic strategy for controlling CRC and other types of cancer with aberrantly activated the Wnt/β-catenin and EGFR-RAS pathways, including those with resistance to EGFR-targeting drugs attributed to *KRAS* mutations.

## Colorectal cancer: Potential treatment for drug-resistant cases

A recently identified small molecule shows promise for tackling resistance to a leading colorectal cancer drug. Three proteins that are over-expressed in colorectal cancer are epidermal growth factor receptor (EGFR), RAS and β-catenin. These proteins and their interconnected signaling pathways are therefore important therapeutic targets. EGFR is the target of the drug cetuximab, but many patients are resistant to this drug attributed to mutations in a gene that influences the signaling pathways of the three key proteins. Kang-Yell Choi at Yonsei University in Seoul, South Korea, and co-workers trialed a novel molecular drug on human colorectal cancer tissues and on mice. They confirmed that the new drug leads to reduced EGFR levels by degrading RAS and β-catenin and therefore suppresses the growth of colorectal cancer cells in samples with or without the resistant mutations.

## Introduction

The Wnt/β-catenin and RAS/extracellular signal-regulated kinase (ERK) pathways play important roles in the regulation of various cellular events, including proliferation, differentiation, and transformation. Mutations in genes in both pathways, such as *adenomatous polyposis coli* (*APC*) and *KRAS*, which occur in as many as 90% and in 40–50%^1,2^, respectively, of human colorectal cancers (CRCs), are known to be involved in the initiation and progression of CRC. Both *APC* and *KRAS* mutations synergistically promote cellular transformation and tumor growth^3–5^, which is attributed to the activation of the RAS-ERK pathway via the aberrant activation of the Wnt/β-catenin signaling caused the loss of *APC*^6–8^. One of the key events in the crosstalk between the two pathways is the stabilization of RAS, especially oncogenic mutant KRAS, as well as β-catenin, caused by *APC* loss. RAS, as well as β-catenin, is degraded via glycogen synthase kinase 3 beta (GSK3β)-mediated phosphorylation and subsequent poly-ubiquitination-dependent proteasomal degradation; however, the inactivation of GSK3β by the loss of *APC* stabilizes these proteins^9,10^.

The epidermal growth factor receptor (EGFR), a transcriptional target of the Wnt/β-catenin pathway^11,12^, is also overexpressed in human CRC and plays a synergistic role with the Wnt/β-catenin pathway in tumorigenesis^13–15^. Therefore, therapies targeting both the Wnt/β-catenin and EGFR-RAS-ERK pathways, especially those lowering the levels of β-catenin, RAS, and EGFR, can be ideal approaches for the treatment of CRC. Based on the identification of the mechanism of RAS destabilization by the Wnt/β-catenin signaling and the large increase in both β-catenin and RAS levels in human CRC, we screened and characterized small molecules that suppress the growth of CRC cells via the destabilization of both β-catenin and RAS^16,17^. KYA1797K, a small molecule derived from the chemical synthesis of an initial hit, directly interacts with the *N*-terminal regulator of G protein signaling (RGS) domain of axin and results in the phosphorylation and subsequent degradation of both β-catenin and RAS via the activation of GSK3β^16^.

The drugs targeting the EGFR-RAS-ERK pathway, such as the anti-EGFR antibody drugs including cetuximab and panitumumab, have been prescribed as therapeutic drugs for patients with cancers such as metastatic CRC. However, these drugs have limitations in their usage due to the poor efficacy or the insensitivity in patients harboring *KRAS* mutations^18–20^, and have no alternative therapy. Therefore, we tested the effects of KYA1797K on the growth and transformation of *KRAS*-mutated CRC cells resistant to cetuximab because KYA1797K reduces both β-catenin and RAS protein levels. KYA1797K suppresses the growth and transformation of CRC cells, reduces tumor xenograft growth and decreases the colony formation and growth of tumor organoids derived from CRC patient tissues insensitive to cetuximab due to *KRAS* mutations via the degradation of oncogenic KRAS and β-catenin with EGFR repression.

Together, small molecules that degrade both β-catenin and RAS via the transcriptional repression of EGFR can be a potential therapy for the treatment of CRC patients with aberrantly activated Wnt/β-catenin and EGFR-RAS pathways attributed to the increase in β-catenin, EGFR, and RAS as well as to their activation by pathologically important *APC, KRAS*, and *EGFR* mutations.

## Materials and methods

### Cell culture and reagents

Colorectal cancer (CRC) cells, including HCT15, RKO, SW480, and DLD1, were purchased from the American Type Culture Collection (ATCC, Manassas, VA, USA). Isogenic human CRC cells, DLD-1 cells harboring wild-type (WT), or mutant (MT) *KRAS* (D-WT and D-MT, respectively)^21^ were provided by B. Vogelstein (John Hopkins University School of Medicine, Baltimore, MD, USA). DiFi cells were provided by Won-Ki Kang (Division of Hematology-Oncology at Samsung Medical Center, Seoul, Republic of Korea). All the cell lines were authenticated using short tandem repeat profiling (Cosmogenetech, Seoul, Republic of Korea). RKO cells were cultured in DMEM (Gibco Life Technologies, Grand Island, NY, USA) supplemented with 10% fetal bovine serum (FBS; Gibco Life Technologies). HCT15, DLD1, SW480, DiFi, D-WT, and D-MT cells were maintained in RPMI 1640 medium (Gibco Life Technologies) supplemented with 10% FBS. For the establishment of stable cell lines, D-WT cells were transfected with Myc-KRAS-WT-pcDNA3.1, Myc-KRAS-G12V-pcDNA3.1, or empty-pcDNA3.1 plasmid, and were selected in a medium containing G-418 (Sigma-Aldrich; St. Louis, MO, USA). For the establishment of the *APC* knockout (KO) cell lines, RKO cells were transfected with the LentiVRISPRv2 vector containing the *APC* gene guide sequence and were selected in a medium containing puromycin (Sigma-Aldrich, St. Louis, MO, USA). Lipofectamine (Invitrogen, Waltham, MA, USA) and Lipofector-EZ (AptaBio, Gyeonggi, Republic of Korea) were used for plasmid transfection according to the manufacturer’s instructions. KYA1797K was dissolved in dimethyl sulfoxide (DMSO) (Sigma-Aldrich) for the in vitro studies. Cetuximab (C225, Erbitux) was obtained from Merck (Darmstadt, Germany).

### Plasmids

The plasmids Myc-APC-pCMV were provided by Eric R. Fearon (University of Michigan, USA). The constructs Myc-WT-KRAS-pcDNA3.1 and Myc-G12V-KRAS-pcDNA3.1 were previously generated^9^. To construct the APC knockout vector using the CRISPR-CAS system, we designed and synthesized the guide oligos targeting the APC gene (upstream of the NGG PAM sequence) using the following sequences: forward, 5′-CACCGTCGCTCTTCATGGATTTTTA-3′; and reverse, 5′-AAACTAAAAATCCATGAAGAGCGAC-3′ (Bioneer, Daejeon, Republic of Korea). Two oligos were annealed and ligated into the *BsmB1*-digested lentiCRISPRv2 vector (Addgene, Cambridge, MA, USA), and transformed into the JM109 bacterial strain. All the constructs and mutations were confirmed by nucleotide sequencing analyses (Cosmogenetech, Daejeon, Republic of Korea).

### Human CRC tissue microarray (TMA) analyses

The normal, polyp/adenoma, and adenocarcinoma samples were analyzed using CRC TMA (Biomax, Inc., Rockville, MD, USA). Immunohistochemical analyses were performed with anti-EGFR, anti-β-catenin, and anti-pan-RAS antibodies. The TMA slides were visualized by microscopy (Eclipse 80i; Nikon, Melville, NY, USA). For quantitative analyses, the H-score of each stained sample was determined by IHC profiler software (Image J) using the following scale: H-score=3×, highly positive population, +2×, positive population, +1×, weak positive population, and +0×, negative population.

### Animal study

All animal experiments were performed in accordance with the Korean Food and Drug Administration guidelines. The protocols were reviewed and approved by the Institutional Animal Care and Use Committee (IACUC) of Yonsei University. C57BL/6J-*Apc*^*min/+*^ (*Apc*^*min/+*^) and B6.129S-*Kras*^*tm3tyj*^ (*Kras*^*G12D*^LA2) mice were obtained from the Jackson Laboratory. To generate *Apc*^*min/+*^*/Kras*^*G12D*^*LA2* mice for the tumor organoid experiment, *Apc*^*min/+*^ mice were crossed with *Kras*^*G12D*^*LA2* mice. For the xenograft study, athymic *nu/nu* mice were injected subcutaneously in the dorsal flank with D-MT (1 × 10^7^ cells/mouse) in 200 µL of PBS: Matrigel^®^ (1:1; BD Biosciences, San Jose, CA, USA). When the mean tumor size was between 100–200 mm^3^, the mice were randomly divided into four groups: vehicle, cetuximab, KYA1797K, or co-treatment with KYA1797K and cetuximab. The tumors were measured using Vernier calipers every 3 days, and the volumes were then calculated according to the following formula: π/6 × length × width × height. Twenty-one days after the drug treatment, the mice were killed, and the tumors were excised and fixed in 4% paraformaldehyde (PFA; Wako, Richmond, VA, USA) or snap frozen in liquid nitrogen for further analyses.

### Tumor organoid experiments

For the human tumor organoid experiments, the use of human tissue was reviewed and approved by the Institutional Review Board of Severance Hospital, Yonsei University College of Medicine. The biopsied tumor samples from CRC patients were cut into 3–5 mm pieces, washed with ice-cold PBS three times, and incubated with collagenase II (2 mg/mL), hyaluronidase (20 µg/mL), and Ly27632 (10 µM) for 1 h at 37 °C. Small intestinal tumors from *Apc*^*Min/+*^*/Kras*^*G12D*^*LA2* mice were isolated and washed with ice-cold PBS, and single cells isolated from the tumors were collected using 0.25% trypsin containing 10 mM Ly27632 and 100 µg/mL Primocin^™^ for 30 min. After incubation, 1 × B27 was added, and the mixture was filtered through 100 µm and 40 µm cell strainers (BD Biosciences, San Jose, CA, USA) to collect single cells. The cells were mixed with growth factor-reduced Matrigel^®^ (BD Bioscience). After the gel solidified, N2 medium containing 10% R-spondin-1 CM, 100 µg/mL noggin, 1.25 mM *N*-acetyl cysteine, 10 mM nicotinamide, 50 ng/mL EGF, 10 nM gastrin, 500 nM A83-01, 3 µM SB202190, and 10 nM prostaglandin E2 was added. The growth medium was refreshed every 2 days, and the cells were passaged by mechanical disruption every 10–14 days at a 1:5 split ratio. The measurements of tumor organoid growth were performed by a CellTiter-Glo^®^ assay (Promega, Madison, WI, USA) according to the manufacturer’s instructions. Luminescence was measured using a FLUOstar Optima instrument (BMG Labtech, Cary, NC, USA).

### Immunoblotting

Immunoblot analysis was performed as previously described^16^. Immunoblotting was performed with the following antibodies: anti-pan-RAS (sc-4), anti-β-catenin (sc-7199), anti-ERK (sc-514302), anti-APC (sc-896), anti-PCNA (sc-56), anti-KRAS (sc-30), and anti-Myc (sc-40) purchased from Santa Cruz Biotechnology; anti-p-ERK (9101) and anti-α-tubulin (3873) obtained from Cell Signaling Technology; and anti-EGFR (ab52894) and anti-p-EGFR (ab5644) purchased from Abcam (Cambridge, United Kingdom).

### GTP-RAS pull-down assays

For the detection of GTP-RAS (active RAS), the GST-Raf1 RAS-binding domain (RBD) fusion protein was purified from cell lysates of transformed BL21 cells using glutathione-agarose beads and then resuspended in GST-FISH buffer [10% glycerol, 50 mM Tris (pH 7.4), 100 mM NaCl, 1% NP-40, and 2 mM MgCl_2_] with a 50% bead slurry. Purified GST-Raf1 RBD proteins were incubated with the WCL of D-WT or D-MT cells treated under the indicated conditions, and the beads were washed with GST-FISH buffer at least five times. The pull-down samples were subjected to immunoblot analyses.

### Cell proliferation assays

For the cell proliferation assays, CRC cells were plated at a density of 2–5 × 10^3^ cells/well in a 96-well plate. The cells were then treated under the indicated conditions for 96 h. After incubation, 3-(4,5-dimethylthiazol-2-yl)-2-5-diphenyltetrazolium bromide (MTT; Amresco, Solon, OH, USA) reagent was added to each well at a concentration of 0.25 mg/mL for 2 h at 37 °C. Insoluble purple formazan was obtained by removing the medium and extracting it with DMSO. The absorbance at 590 nm was monitored with the FLUOstar Optima microplate reader (BMG Labtech).

### Colony formation assays

CRC cells were seeded in 12-well plates (250–600 cells/well). The cells were treated under the indicated conditions for 7–16 days with changes of the medium every 4 days. At the end of the experiment, the cells were washed, fixed, and stained with 0.5% crystal violet in 20% ethanol for 2 h, and subsequently washed three times with distilled water.

### Reverse transcription and quantitative real-time PCR

The total RNA was isolated using TRIzol^®^ reagent (Invitrogen, Waltham, MA, USA) according to the manufacturer’s instructions. The total RNA (2 µg) was reverse transcribed using 200 units of M-MLV reverse transcriptase (Invitrogen) in a 20 µL reaction mixture at 42 °C for 1 h. For quantitative real-time PCR analyses, the resulting cDNA (1 µL) was amplified in 10 µL of‘ Rotor-gene SYBR^®^ green (Qiagen, Hilden, Germany). The comparative cycle threshold (C_T_) method was used, and *β-actin* served as an endogenous control. The primers are listed in the supplementary table S1.

### Immunohistochemistry

Immunohistochemistry (IHC) was performed as previously described^17^. For peroxidase IHC analysis, sections were incubated with the primary antibody overnight at 4 °C, followed by incubation with biotinylated anti-mouse (Vector Laboratories, Burlingame, CA, USA) or biotinylated anti-rabbit (Vector Laboratories) secondary antibodies at RT for 1 h. The samples were incubated in the avidin-biotin complex solution (Vector Laboratories), they were then stained with 3, 3′ diaminobenzidine (DAB; Vector Laboratories) for 5 min and counterstained with Mayer’s hematoxylin (Muto, Tokyo, Japan). The signals were analyzed using a bright field microscope (Nikon TE-2000U). The fluorescence signal was visualized using a confocal microscope (LSM510; Carl Zeiss, Oberkochen, Germany) at excitation wavelengths of 488 nm (Alexa Fluor^®^ 488), 543 nm (Alexa Fluor^®^ 555), and 405 nm (DAPI). At least three fields of view per section were analyzed. The IHC images were quantified from the representing images using an IHC profiler in the ImageJ software.

### Immunocytochemistry

Cells were seeded on collagen-coated (500 µg/mL) coverslips. The cells were then fixed with 5% formalin for 30 min, permeabilized with 0.1% Triton X-100 for 20 min, and preblocked with PBS containing 5% bovine serum albumin (BSA; Affymetrix, Santa Clara, CA, USA) and 1% normal goat serum (NGS; Vector Laboratories) for 1 h. The cells were then incubated with the indicated primary antibody overnight at 4 °C, followed by Alexa Fluor 488 (Life Technologies, Carlsbad, CA, USA) or Alexa Fluor 555 (Life Technologies) secondary antibodies for 4 h at 4 °C and counterstained with DAPI for 10 min at room temperature. After incubation, the cells were mounted in Gel/Mount media (Biomeda Corporation, Foster City, CA, USA). The fluorescence signal was visualized using a confocal microscope (LSM510; Carl Zeiss) at excitation wavelengths of 488 nm (Alexa Fluor^®^ 488), 543 nm (Alexa Fluor^®^ 555), and 405 nm (DAPI).

### Mutational analyses of human CRC patient samples

To analyze the mutational status of both *APC* (chr5, NM_000038.5) and *KRAS* (chr12, NM_033360.3) in the CRC patient samples, total genomic DNA was isolated from the tumor organoids and analyzed by whole exome sequencing (Macrogen).

### Statistical analysis

All the data are represented as the means ± standard deviations of at least three independent experiments. The statistical significance was assessed using the Student’s *t-*test. The significance was denoted as ^*^P<0.05, ^**^P<0.01, and ^***^P<0.001.

## Results

### The levels of β-catenin, RAS, and EGFR are increased in CRC patient tissues

**Fig. 1:**
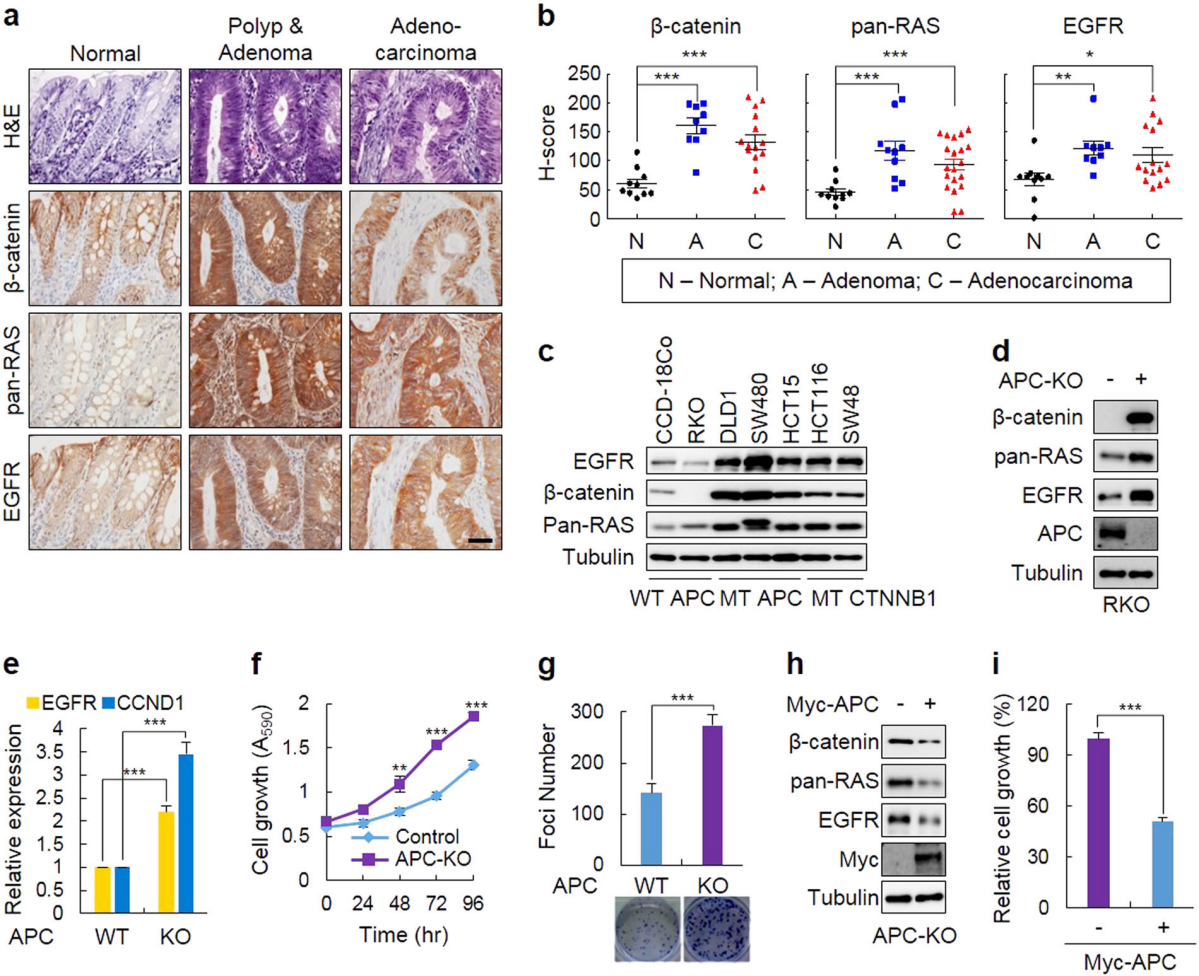
The levels of β-catenin, RAS, and EGFR are increased by APC mutations and correlate with the growth and transformation of CRC cells. **a** Immunohistochemical (IHC) analysis of β-catenin, RAS, or EGFR in the CRC patient tissue microarrays (TMAs) containing normal, polyp/adenoma, and adenocarcinoma. Scale bar, 50 µm. **b** The quantitative analyses of positive signals in the IHC images were performed by comparing the H-scores of staining for β-catenin, RAS, or EGFR in normal tissues and at different stages of colorectal tumorigenesis. Normal mucosa (*n* = 10), polyp/adenoma (*n* = 9), and adenocarcinoma (*n* = 15). **c** Immunoblot (IB) analysis to detect β-catenin, RAS, and EGFR in various CRC cell lines harboring different mutations of *APC* or *CTNNB1*. **d–g** The effects of APC knockout (KO) on the expression of β-catenin, RAS or EGFR and on cell growth/transformation. The APC-KO RKO cells and RKO cells expressing the control vector alone were grown, and IB analyses were performed with whole cell lysates (WCLs) to detect each protein (**d**). Quantitative polymerase chain reaction (qPCR) assays were performed to determine the expression of the *EGFR* and *CCND1* mRNAs (**e**). 3-(4,5-Dimethylthiazol-2yl)-2-5-diphenyltetrazolium bromide (MTT) assays (**f**) were performed for measurements of cell growth, and foci formation assays (**g**) were performed to detect cellular transformation. **h**, **i** The effects of overexpression of APC. The APC-KO RKO cells were transfected with Myc-tagged APC for 36 h, and the WCLs were subjected to IB analysis (**h**). The relative cell growth was measured by the MTT assay (**i**). The data are presented as the mean ± SD. Two-sided Student’s *t-*test, ^*^P<0.05, ^**^P<0.005, and ^***^P<0.001

Given that both β-catenin and RAS are stabilized in CRC patient tissues mostly having *APC* mutations and that EGFR is a transcriptional target of the Wnt/β-catenin pathway, we tested whether expression levels of β-catenin, RAS, and EGFR are correlated in CRC patient tissues. The levels of the EGFR were higher along with β-catenin and pan-RAS, especially in the polyp/adenoma and adenocarcinoma regions of the CRC patient tissues, compare with paired normal tissues (Fig. 1a, b). Similar correlated increases of the proteins were observed in *APC-* or *CTNNB1* (encoding β-catenin)-mutated CRC cell lines (Fig. 1c). In addition, two independent CRC cohorts from the oncomine database indicated that the mRNA level of EGFR in the CRC tissues including adenoma, carcinoma, or adenocarcinoma is higher than that in the normal colon tissues (Supplementary Figure S1). These results are correlated with the protein level pattern of EGFR in the CRC patient tissue microarrays (TMAs). To further confirm these increases in the β-catenin, pan-RAS, and EGFR levels by pathologically meaningful *APC* loss in CRC, we generated RKO cells with the knockout (KO) of *APC*. The levels of β-catenin, pan-RAS, and EGFR were simultaneously increased (Fig. 1d), and the mRNA levels of the *EGFR* and *CCND1* (cyclin D1) genes, both well-known Wnt/β-catenin signaling response genes, were increased in the *APC*-KO RKO cells (Fig. 1e). Furthermore, the growth and transforming abilities of the *APC*-KO RKO cells were significantly elevated compared with those of the RKO cells (Fig. 1f, g). In addition, all of the *APC* knockout effects were rescued by the overexpression of APC in *APC*-KO RKO cells (Fig. 1h, i).

The Wnt/β-catenin pathway-dependent co-regulation of EGFR and RAS was also confirmed by the increased levels of these proteins induced by recombinant Wnt3a treatment in both HEK293 and RKO cells harboring wild-type (WT) *APC* and *CTNNB1* (Supplementary Figure S2a) and by the decreased levels of these proteins induced by the knockdown of β-catenin in both HCT15 and DLD1 cells harboring *APC* mutations (Supplementary Figure S2b). The mRNA levels of *EGFR* and *CCND1* were increased and decreased by Wnt3a treatment and β-catenin knockdown, respectively (Supplementary Figure S2c and d). Therefore, EGFR and β-catenin/RAS are increased by the loss of *APC* in CRC cells at both the transcriptional and protein levels.

### The stabilization as well as the mutation of RAS contributes to the cetuximab resistance of CRC cells

**Fig. 2:**
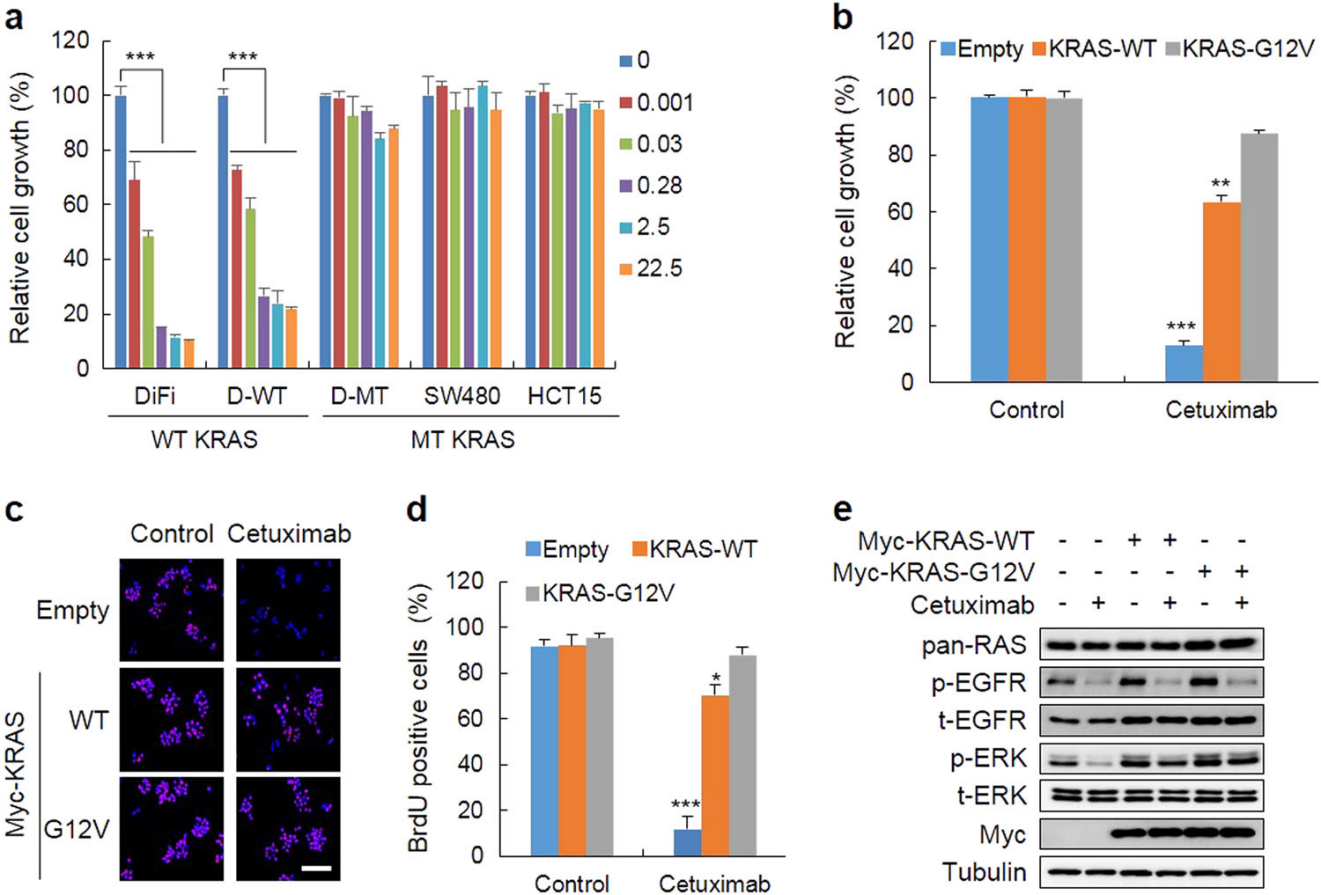
The overexpression of WT KRAS, as well as the mutant form, contributes to the resistance to cetuximab. **a** MTT assays were performed to determine the effects of cetuximab on the growth of various CRC cells treated with the indicated doses of cetuximab for 96 h. **b–e** The effects of the overexpression of KRAS WT or oncogenic mutant G12V on cell growth. The D-WT cells stably expressing Myc-KRAS-WT, -G12V, or empty vector were treated with 5 µg/mL cetuximab for 96 h. MTT (**b**) and bromodeoxyuridine (BrdU) incorporation (**c**, **d**) assays were performed to measure cell proliferation. Scale bar, 100 µm. The WCLs were subjected to IB analyses (**e**). The data are presented as the mean ± SD (*n* = 3). Two-sided Student’s *t-*test, ^*^P<0.05, ^**^P<0.005, and ^***^P<0.001

Although drugs targeting the EGFR, such as cetuximab, have been used for the treatment of CRC, their efficacies were limited because the drugs were ineffective for the treatment of patients having *KRAS* mutations^18^^–^^20^. Similarly, the growth of *KRAS*-mutated D-MT, HCT15, and SW480 cells was not significantly inhibited, while the growth of DiFi and D-WT cells harboring WT *KRAS* was effectively inhibited by cetuximab (Fig. 2a).

The resistance to cetuximab in CRC cells with *KRAS* mutations was further investigated by using D-WT cells, a DLD-1-derived cell line that harbors only WT *KRAS*^21^. Although 90% of the growth of D-WT cells was inhibited by cetuximab treatment for 96 h, only 10% of the growth was inhibited in cells overexpressing MT (G12V)-KRAS (Fig. 2b). Interestingly, cetuximab resistance was also observed for the overexpression of WT KRAS, although the degree of resistance was somewhat lower (65%) (Fig. 2b). These results were similar to the inhibitory patterns of cell proliferation measured by the bromodeoxyuridine (BrdU) incorporation assays (Fig. 2c, d). The patterns of antiproliferation induced by KRAS overexpression correlated with the ERK activity in the cells treated with cetuximab (Fig. 2e). Overall, the resistance to the EGFR inhibitor cetuximab affecting CRC cell growth was due to the increase in the level as well as the mutation of *KRAS*.

### KYA1797K, a small molecule that degrades both β-catenin and RAS, overcomes cetuximab resistance in CRC

**Fig. 3:**
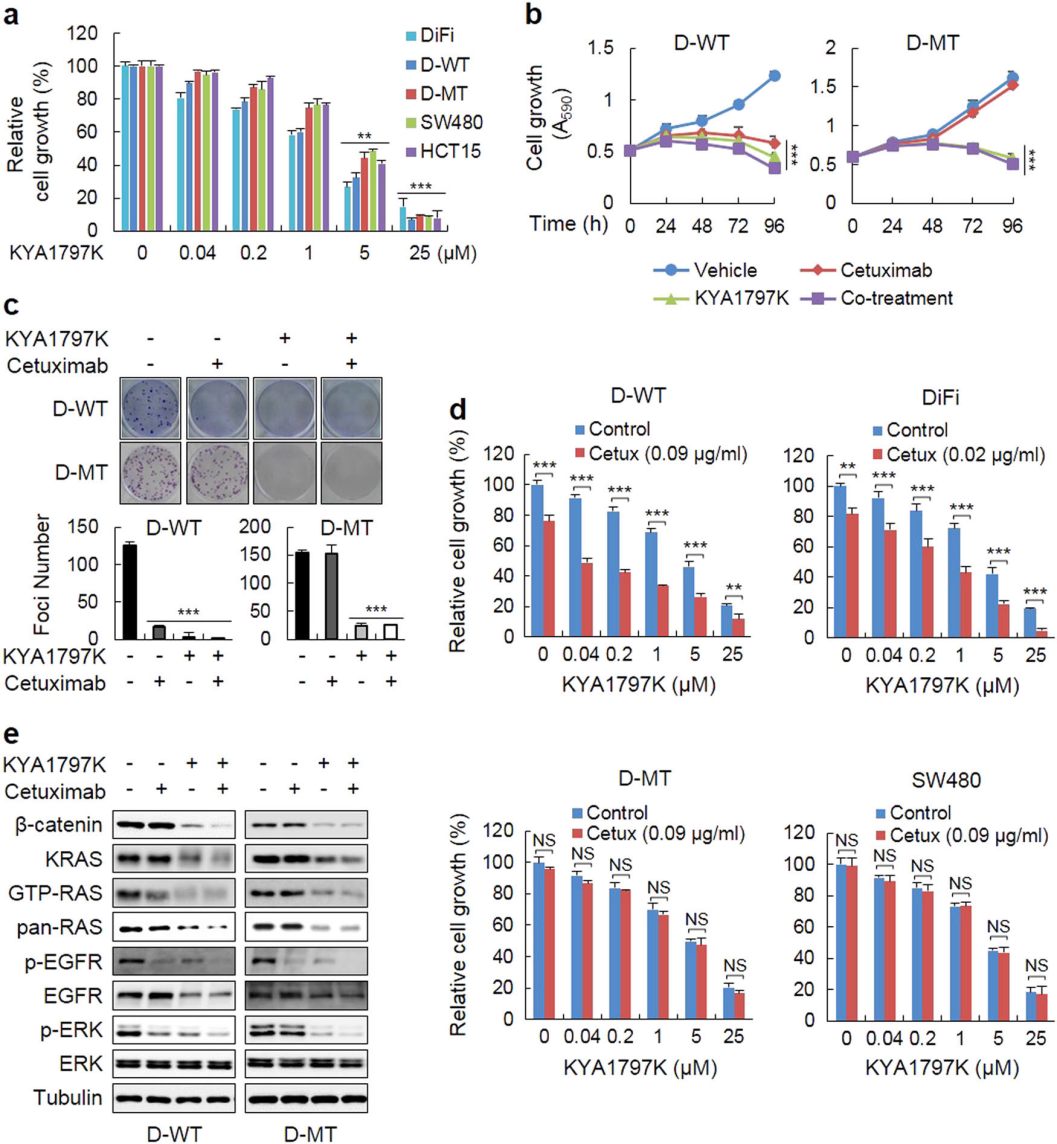
KYA1797K overcomes the resistance to cetuximab attributed to *KRAS* mutations on the growth and transformation of CRC cells. **a** MTT assays were performed to determine the effects of KYA1797K on the growth of various CRC cells treated with the indicated doses of KYA1797K for 96 h. **b**, **c** The D-WT or D-MT cells were treated with vehicle, 25 µM KYA1797K, 5 µg/mL cetuximab, or were co-treated with both for the indicated time periods. The growth and transformation of the D-WT or D-MT cells were monitored by MTT (**b**) and foci formation (**c**) assays, respectively. **d** MTT assays were performed to determine the combined effect of KYA1797K and cetuximab in D-WT, DiFi, D-MT, and SW480 cells co-treated with the indicated doses of KYA1797K and cetuximab for 96 h. **e** IB analysis of D-WT or D-MT cells treated under the indicated conditions for 24 h. Glutathione-S-transferase (GST)-Raf-1 RAS-binding domain (RBD) pull-down assays were performed to detect active RAS (GTP-RAS). The data are presented as the mean ± SD (*n* = 3). Two-sided Student’s *t-*test, ^**^P<0.005, and ^***^P<0.001. NS not significant

Because the increase in β-catenin, RAS, and EGFR levels in CRC contributes to synergistically promote cell growth and transformation, we tested the effects of KYA1797K on the growth of CRC cells, especially cells resistant to cetuximab, due to *KRAS* mutations. KYA1797K dose-dependently inhibited the growth of various CRC cells regardless of their *KRAS* mutational status (Fig. 3a), while the growth of CCD18-Co, normal colon cell, was mildly affected by KYA1797K treatment (Supplementary Figure S3). The effectiveness of KYA1797K but not cetuximab on the inhibition of the growth and transformation of cells harboring *KRAS* mutations was confirmed by comparing WT- and MT-*KRAS* CRC cells (Fig. 3b, c; Supplementary Figure S4a and b). Co-treatment with cetuximab and KYA1797K cumulatively inhibited the growth of D-WT cells but not D-MT cells (Fig. 3b). The differential effects of co-treatment with KYA1797K and cetuximab on the growth of CRC cells, which are dependent upon the mutational status of *KRAS*, were also observed in a dose-dependent manner in WT *KRAS* D-WT and DiFi cells but not in the *KRAS-*mutated D-MT and SW480 cells (Fig. 3d).

The inhibition of ERK activity, as well as GTP-RAS activity, by treatment with cetuximab or KYA1797K alone or in combination in CRC cells with different *KRAS* mutations was correlated with cell growth and transformation (Fig. 3e and Supplementary Figure S4c). The levels of β-catenin, RAS, and EGFR were reduced by KYA1797K but not by cetuximab although the p-EGFR levels were reduced by both cetuximab and KYA1797K treatment in WT and MT *KRAS* cells. Consistently, the EGFR mRNA levels were reduced without a change in the β-catenin mRNA levels by KYA1797K but not by cetuximab alone (Supplementary Figure S5). Overall, it was shown that KYA1797K degrades both β-catenin and RAS proteins with the transcriptional repression of EGFR. Treatment with KYA1797K overcame the ineffectiveness of cetuximab for inhibiting the colony formation ability and growth of CRC cells harboring *KRAS* mutations.

### KYA1797K overcomes the resistance to cetuximab attributed to *KRAS* mutations in tumor xenografts

**Fig. 4:**
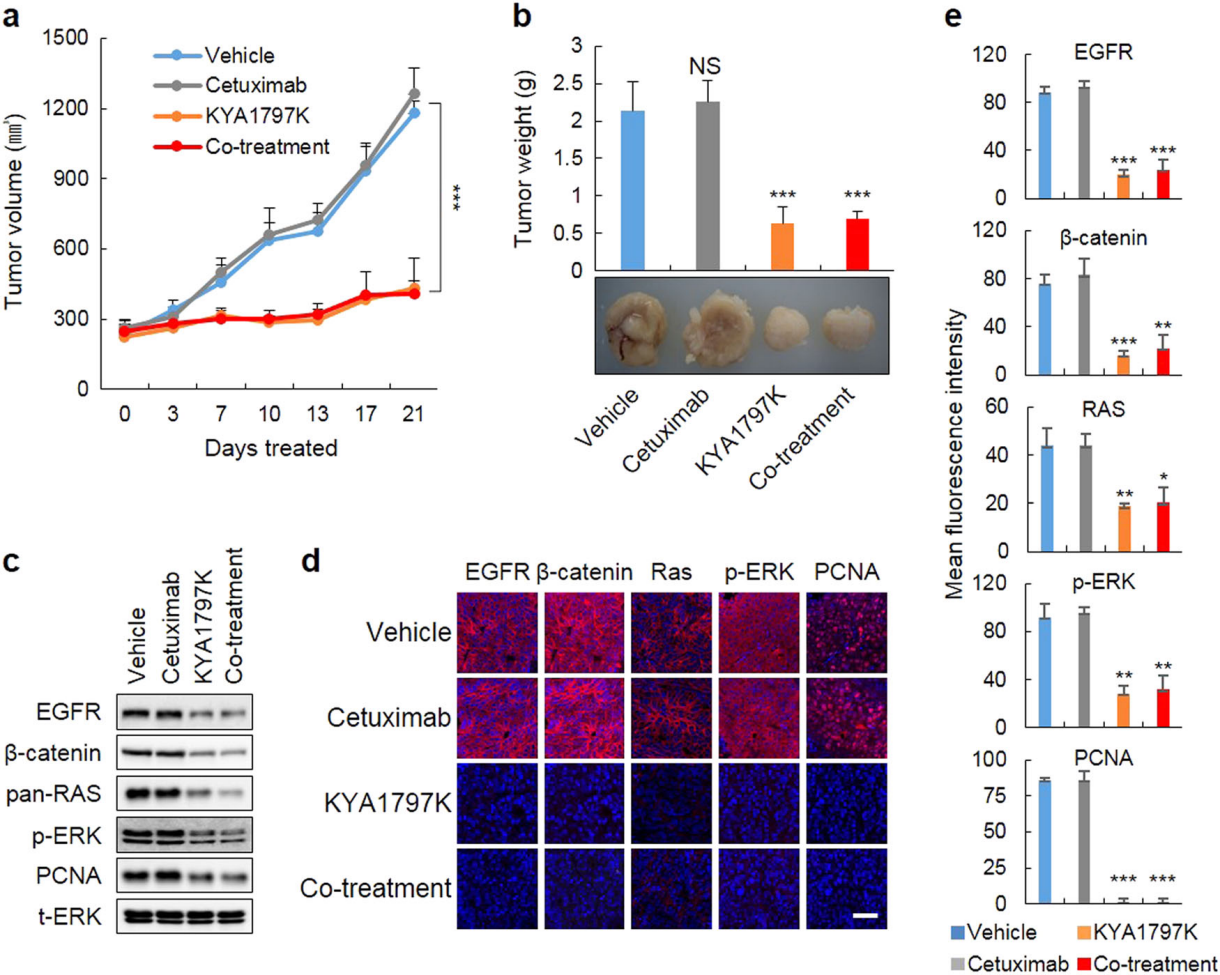
KYA1797K overcomes the resistance to cetuximab attributed to *KRAS* mutations on tumor xenograft growth. **a** D-MT cells were subcutaneously injected into nude mice with the subsequent intraperitoneal (i.p.) injection of vehicle, 20 mg/kg KYA1797K, 1 mg/mouse cetuximab, or a combination of KYA1797K (20 mg/kg) and cetuximab (1 mg/mouse) for 21 days. The tumor volumes were measured every 3 days. **b** The measurement of tumor weights (**b**, upper panel) and the visualization of tumor images (**b**, lower panel) were performed at the time of killing. **c** WCLs prepared from the tumor tissues were subjected to IB analysis. **d** IHC analysis of the tissue sections incubated with the indicated antibodies and then counterstained with 4′,6-diamidino-2-phenylindole (DAPI). Scale bar, 50 µm. **e** The levels of the proteins were measured by the mean fluorescence intensity. The data are presented as the mean ± SD (*n* = 5). Two-sided Student’s *t-*test, ^*^P<0.05, ^**^P<0.005, and ^***^P<0.001. NS not significant

The effectiveness of KYA1797K in the cetuximab-resistant CRC cells harboring *KRAS* mutations was also tested in vivo using a xenograft mouse model that produced tumors after the implantation of D-MT cells harboring both *APC* and *KRAS* mutations. Mice intraperitoneally (i.p.) injected with cetuximab did not show an effect in tumor growth, whereas mice treated with KYA1797K alone or co-treated with KYA1797K and cetuximab showed a significantly reduced tumor volume and weight (Fig. 4a, b). In agreement with the results of the in vitro studies, treatment with KYA1797K alone or in combination with cetuximab but not cetuximab alone reduced ERK activity and reduced β-catenin, RAS, EGFR, and proliferating cell nuclear antigen (PCNA) protein levels, as shown by the immunoblots (Fig. 4c), immunohistochemical analyses, and subsequent quantification (Fig. 4d, e). Together, these results indicate that KYA1797K efficiently suppressed the growth of tumors and overcame the insensitivity to cetuximab in the *KRAS*-mutated cells via degradation of the KRAS protein.

### KYA1797K suppresses the growth of cetuximab-resistant tumor organoids derived from *Apc*^*Min/+*^*/ Kras*^*G12D*^*LA2* mice or CRC patients harboring mutant *KRAS*

**Fig. 5:**
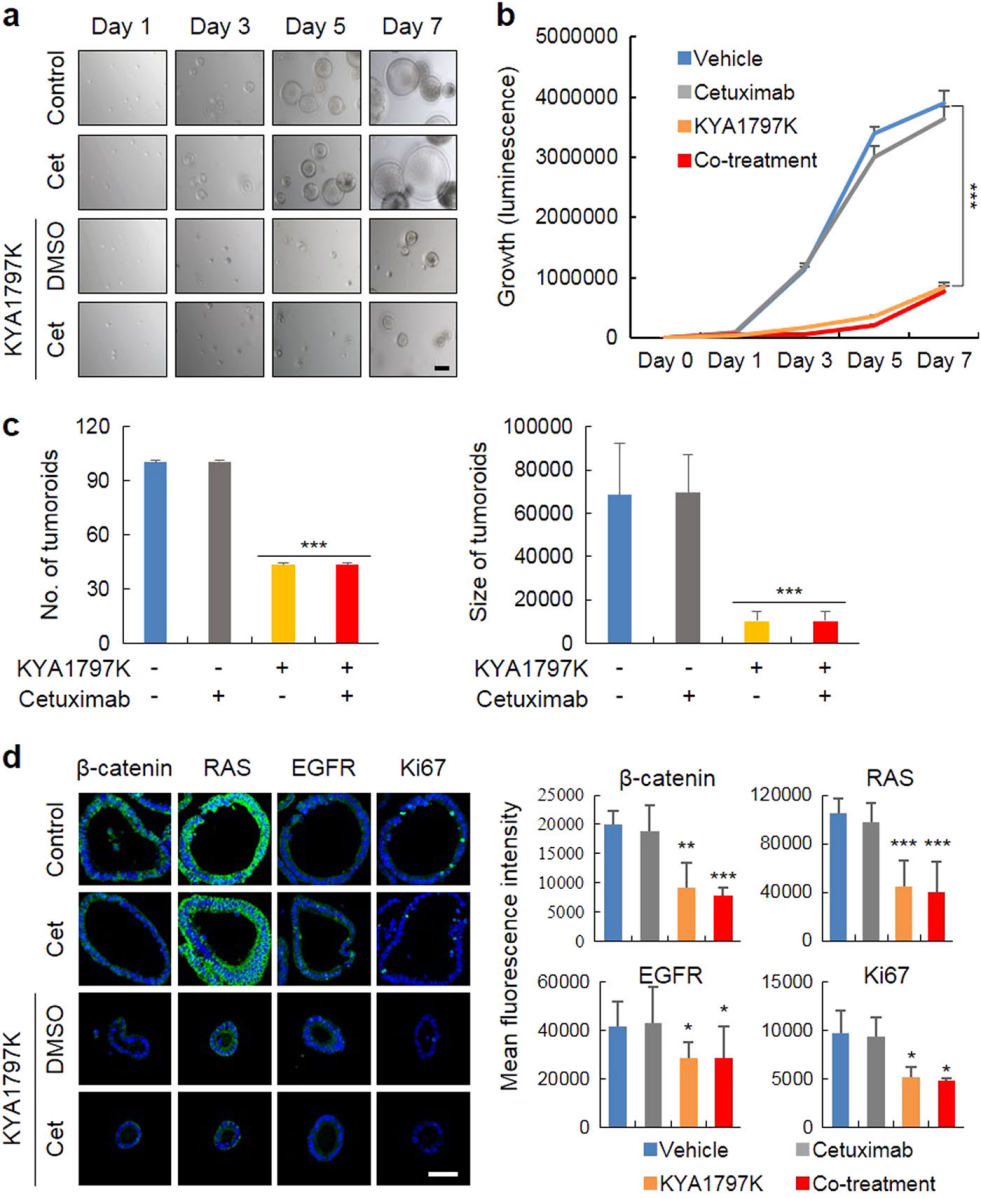
The effectiveness of KYA1797K on the formation and growth of tumor organoids from the small intestine of *Apc*^*Min/+*^*/Kras*^*G12D*^*LA2* mice. The tumor organoids from the small intestine of *Apc*^*Min/+*^*/Kras*^*G12D*^*LA2* mice were cultured as described in the materials and methods section. The tumor organoids were cultured and treated under the indicated conditions for 7 days. **a**, **b** The images of the tumor organoids were visualized (a, scale bar, 200 µm), and the growth of the tumor organoids was measured by a cell titer assay (**b**). **c** The numbers (left) and sizes (right) of the tumor organoids were quantified. **d** Immunocytochemical (ICC) analysis of β-catenin, pan-RAS, EGFR, and Ki67 in the tumor organoids treated under the indicated conditions for 7 days. The tumor organoids were immobilized, immunostained with each antibody, and counterstained with DAPI (**d**, left panel). Scale bar, 50 µm. The levels of these proteins were measured by the mean fluorescence intensity (**d**, right panel). The data are presented as the mean ± SD (*n* = 3). Two-sided Student’s *t-*test, ^*^P<0.05, ^**^P<0.005, and ^***^P<0.001

To further validate the effectiveness of KYA1797K but not cetuximab, we adapted a tumor organoid culture system. Similar to the results of the tumor xenograft studies, both the formation and growth of the tumor organoids from the small intestine of *Apc*^*Min/+*^*/Kras*^*G12D*^*LA2* mice having both *Apc* and *Kras* mutations were specifically suppressed by KYA1797K (Fig. 5a, b). Therefore, both the numbers and sizes of the tumor organoids were significantly reduced by treatment with KYA1797K, while no change was observed for treatment with cetuximab (Fig. 5c). The levels of β-catenin, RAS, EGFR, and Ki-67 were similarly reduced by KYA1797K but not by cetuximab (Fig. 5d). Consistent with the results using the *KRAS-*mutated CRC cell lines, the combined effects of KYA1797K and cetuximab on the growth inhibition were not observed in the tumor organoids derived from the small intestinal tumor cells of *Apc*^*Min/+*^*/Kras*^*G12D*^*LA2* mice.

**Fig. 6:**
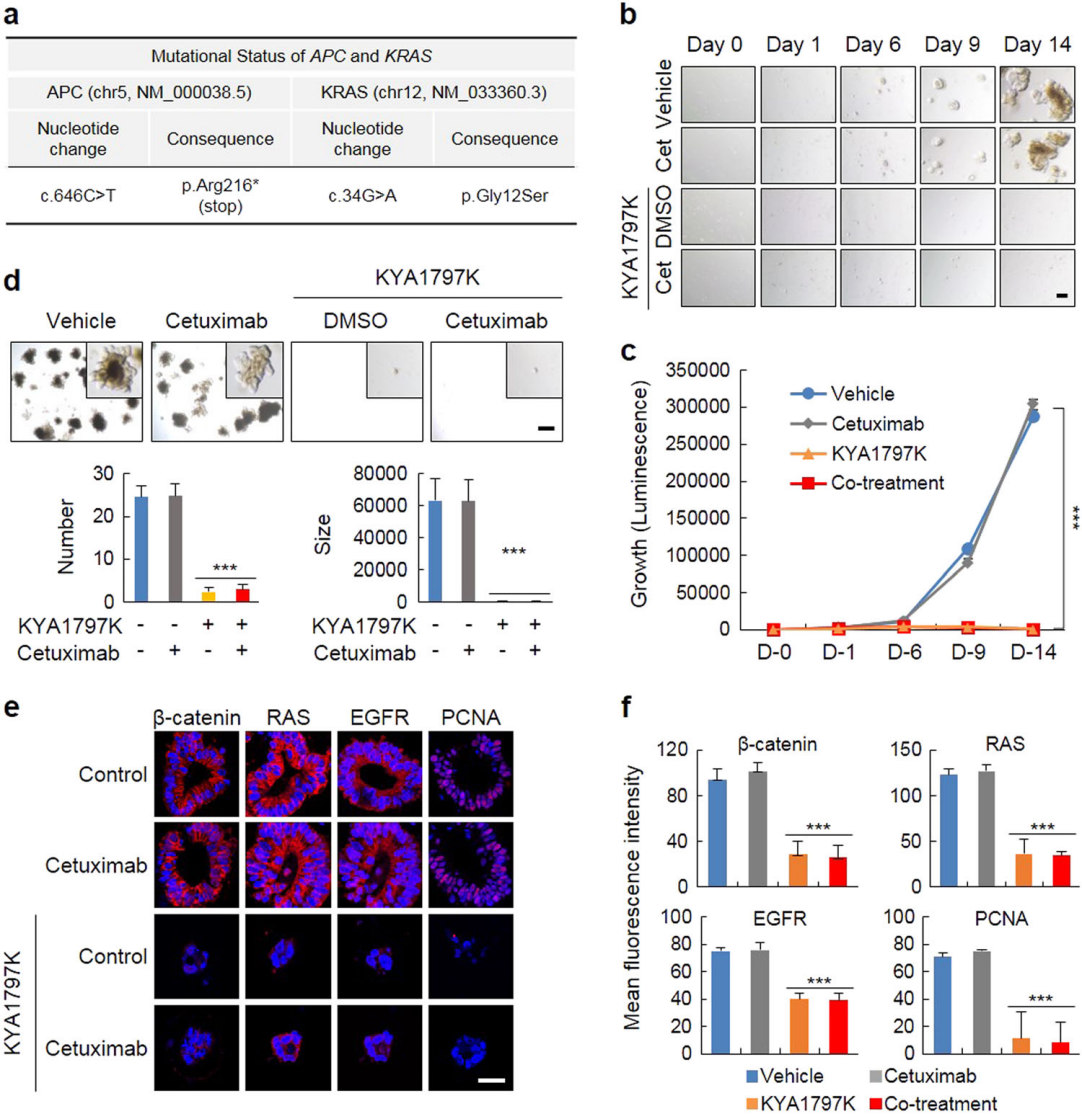
KYA1797K effectively suppresses the formation and growth of tumor organoids derived from CRC patients having both* APC* and *KRAS* mutations. **a** The tumor organoids derived from the CRC patient tissue were cultured as described in the materials and methods section and analyzed by whole exome sequencing to determine the mutational status of the genes of interest, including *KRAS* and *APC*. **b**, **c** The tumor organoids were treated under the indicated conditions for 14 days. The tumor organoids were visualized (**b**, scale bar, 200 µm), and the growth of the tumor organoids was measured by a cell titer assay (**c**). **d** Images of the tumor organoids treated with the indicated conditions for 21 days were captured at low magnification (upper panel, scale bar, 1 mm), and high-magnification images were visualized in the boxed region (inset). The number and size of the tumor organoids were quantified (**d**, lower panel). **e** ICC analysis of β-catenin, pan-RAS, EGFR, or PCNA in the tumor organoids cultured under the indicated conditions for 14 days. The tumor organoids were immobilized and then immunostained with antibodies. Scale bar, 50 µm. **f** The levels of these proteins were measured by the mean fluorescence intensity. The data are presented as the mean ± SD (*n* = 3). Two-sided Student’s *t-*test, ^***^P *<* 0.001

To test the potential application of KYA1797K in humans, we established tumor organoids derived from CRC patient tissues harboring both *APC* (truncated form, Arg216*stop) and *KRAS* (Gly12Ser) mutations, which were identified by whole exome sequencing (Fig. 6a). The human tumor organoids were well-developed with gland formation in a time-dependent manner, which recapitulates tumor heterogeneity containing different cell populations^22^. However, KYA1797K effectively inhibited gland formation in tumor organoids (Supplementary Figure S6). Although cetuximab was not effective, the formation and growth of tumor organoids were mostly abolished by KYA1797K (Fig. 6b, c). Therefore, the number and size of the tumor organoids were significantly reduced by treatment with KYA1797K, while no change was shown for treatment with cetuximab (Fig. 6d). The inhibitory effects of KYA1797K on the tumor organoids correlated with the reduction in the β-catenin, pan-RAS, EGFR, and PCNA levels (Fig. 6e, f). Overall, KYA1797K effectively suppressed the formation and growth of tumor-derived organoids from CRC patients and overcame the insensitivity to cetuximab attributed to *KRAS* mutations by reducing the protein levels via degradation.

## Discussion

Cancer development is a dynamic and multistep process involving multiple genetic changes and molecular alterations^23,24^. In CRC, mutations of genes in the Wnt/β-catenin and RAS/ERK pathways, such as *APC* and *KRAS*, synergistically promote transformation^9,25^. Aberrant activation of the Wnt/β-catenin and RAS/ERK pathways by the co-stabilization of β-catenin and RAS as well as by genetic mutations in these pathway components contribute to colorectal tumorigenesis. EGFR is also overexpressed in human CRC and plays a role in synergistic tumorigenesis^14^.

Therefore, the inhibition of both the Wnt/β-catenin and EGFR-RAS-ERK pathways, especially by reducing the levels of the proteins elevated in CRC, can be an ideal approach for the treatment of human CRC. However, a clinically applicable drug that inhibits both the Wnt/β-catenin and EGFR-RAS-ERK pathways or even the Wnt/β-catenin pathway alone is not available. Small molecules such as KYA1797K reduce the levels of β-catenin, RAS, and EGFR and are potential drug candidates to resolve the problem of resistance to EGFR-targeting therapy attributed to *KRAS* mutations. The effectiveness of KYA1797K on the inhibition of cetuximab-resistant *KRAS-*mutated CRC cells is shown by both in vitro and in vivo studies using the mouse xenograft tumor model generated from CRC cells harboring *KRAS* mutations. The effectiveness and potential application of this compound in patients are demonstrated by the reduction in these proteins and the suppression of growth in tumor organoids derived from CRC patient tissues harboring both *APC* and *KRAS* mutations.

The small molecule drugs that reduce the levels of β-catenin, RAS, and EGFR are highly effective for the treatment of CRC because of the elevated protein levels caused by the loss of *APC*, which is observed in ~90% of human CRCs. The approach to controlling CRC by the specific degradation of the β-catenin and RAS proteins can be further supported by a recent study suggesting that the degradation of specific oncogenic proteins is an ideal approach for cancer therapy^26^. Our approach to controlling RAS, especially oncogenic mutant KRAS, via its degradation provides an alternative approach for the development of a clinically applicable drug targeting the undruggable RAS^27–30^. Although β-catenin- and RAS-destabilizing compounds induced the degradation of HRAS and NRAS, these could be beneficial for the treatment of cancer, because the overexpression of WT-HRAS or -NRAS is known to play roles in the promotion of MT-KRAS-driven tumorigenesis^31^. The importance of the additional *KRAS* mutations in colorectal tumorigenesis with *APC* mutations is shown by the role of MT KRAS in the strong secondary activation of Wnt/β-catenin signaling, which involves cancer stem cell activation by a positive feedback loop via the activation of ERK after the initial activation of the pathway caused by the loss of *APC*^25^.

In summary, we show that the levels of β-catenin, RAS, and EGFR are upregulated in a correlated manner by the loss of *APC*, and increases in these proteins critically promote colorectal tumorigenesis. Therefore, small molecules that degrade both β-catenin and RAS with the reduction of *EGFR* transcription can be potential drug candidates for the treatment of CRC, including CRCs that are resistant to the EGFR-targeting therapies because of *KRAS* mutations.

## Electronic supplementary material

Supplementary Information
