# Supplementary material for: A small molecule approach to degrade RAS with EGFR repression is a potential therapy for KRAS mutation-driven colorectal cancer resistance to cetuximab
Source: Exp Mol Med. 2018 Nov 20;50(11):153. doi: 10.1038/s12276-018-0182-2 (PMC6244225; doi:10.1038/s12276-018-0182-2)
Supplement: Supplementary file 1 — Supplementary Information [file 12276_2018_182_MOESM1_ESM.pdf]

Supplementary Information

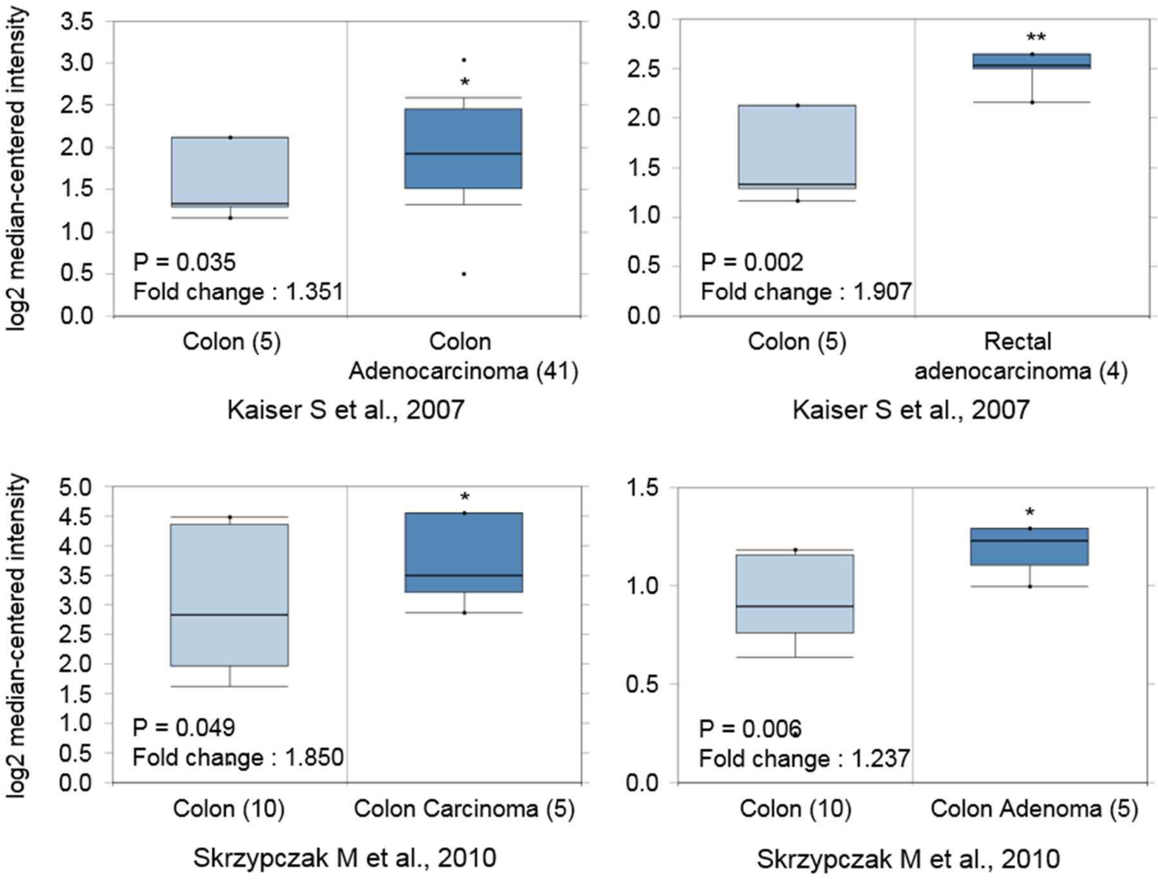

Supplementary Figure S1 Expression of *EGFR* mRNA in human colorectal cancer. Clinical significances of *EGFR* mRNA expression were examined using the oncomine database (www.oncomine.org). Comparison of *EGFR* mRNA levels in two independent datasets between human normal colon tissues (left plot) versus different stage of CRC including adenoma, carcinoma, and adenocarcinoma (right plot). Individual *p*-values and fold changes are indicated within each box plot, $^{*}P < 0.05$ and $^{**}P < 0.005$.

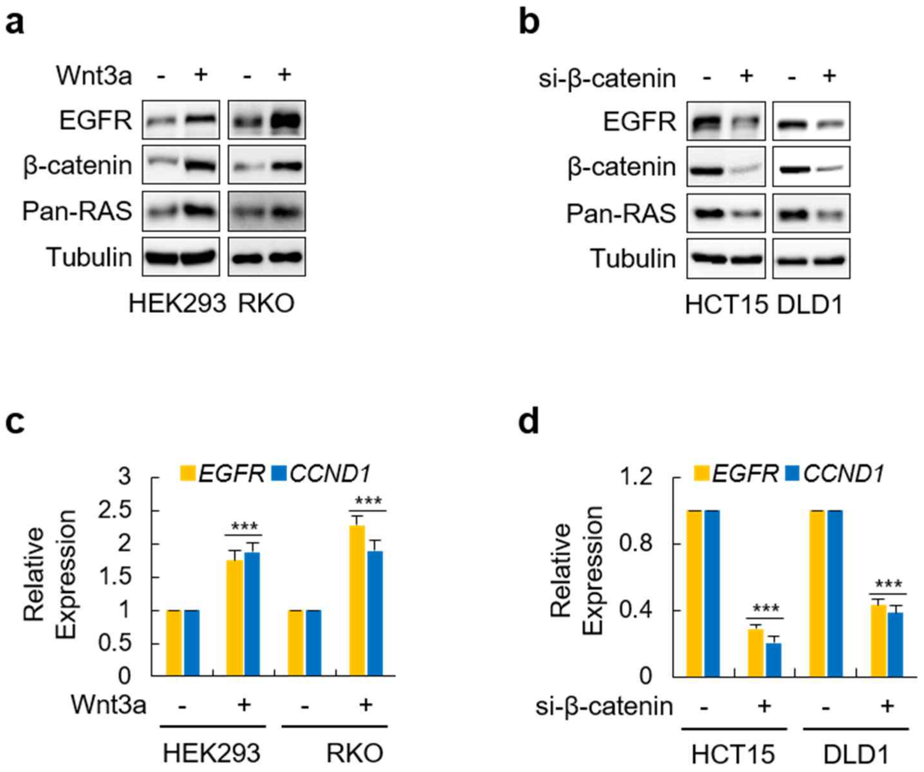

Supplementary Figure S2 The level of EGFR is transcriptionally regulated by the Wnt/β-catenin signaling pathway. The effects of Wnt3a treatment or β-catenin knockdown on the protein and mRNA levels of *EGFR* and *CCND1*. Immunoblots (**a**) and real-time qPCR (**c**) analyses of HEK293 or RKO cells treated with Wnt3a for 24 hr. Immunoblots (**b**) and real-time qPCR (**d**) analyses of HCT15 or DLD1 cells transfected with β-catenin or control siRNA for 48 hr. Data are represented as mean ± SD (n = 3). Two-sided student's t test, $^{***}P < 0.001$.

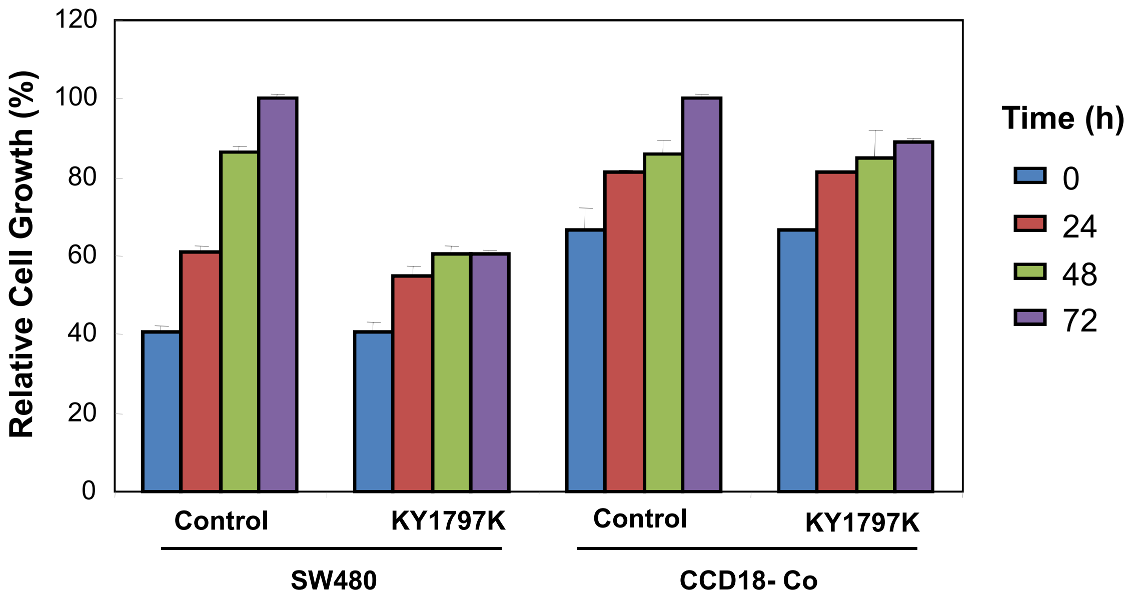

Supplementary Figure S3 The effect of KY1797K on the growth of tumor cells and normal cells. CCD18-Co or SW480 cells were treated with KY1797K (25 μM) or Control. Cell growth was measured by MTT assay at the indicated time points and normalized to the observed value of the respective control at 72 h. The relative cell growth (%) is plotted as the mean ± s.d. ($n = 3$).

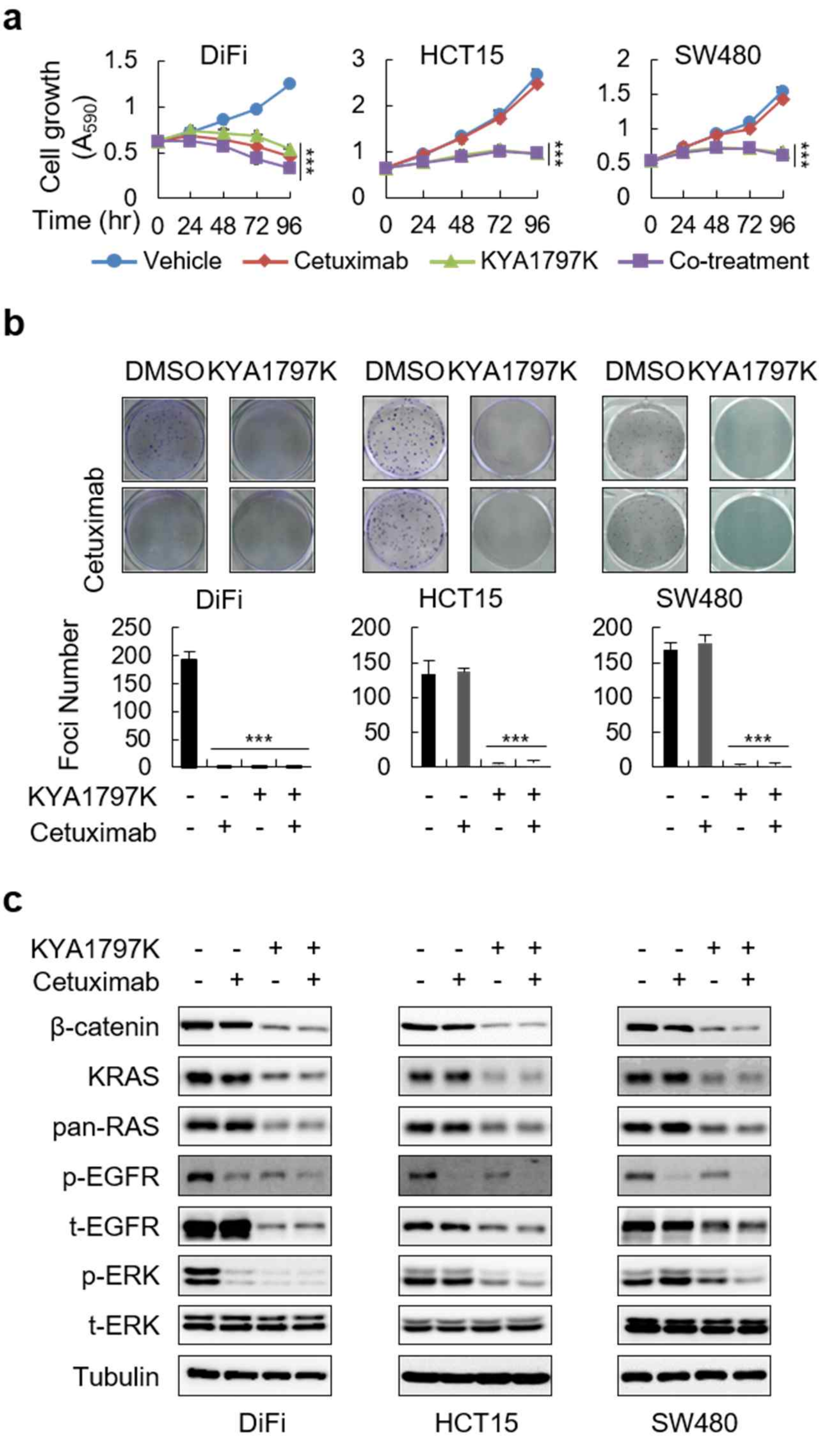

Supplementary Figure S4 The effects of KYA1797K or cetuximab on cell growth and transformation in WT- and MT-*KRAS* CRC cells. DiFi, HCT15, or SW480 cells were treated with vehicle, 20 μM of KYA1797K, 5 μg/mL of cetuximab, or a co-treatment of both for the indicated time periods. MTT assays (**a**) were performed for measurements of cell growth, and

foci formation assays (**b**) were performed to detect cellular transformation. WCLs were subjected to IB analyses to detect each proteins (**c**). Data are represented as mean ± SD ($n = 3$). Two-sided student's t test, $^{***}P < 0.001$.

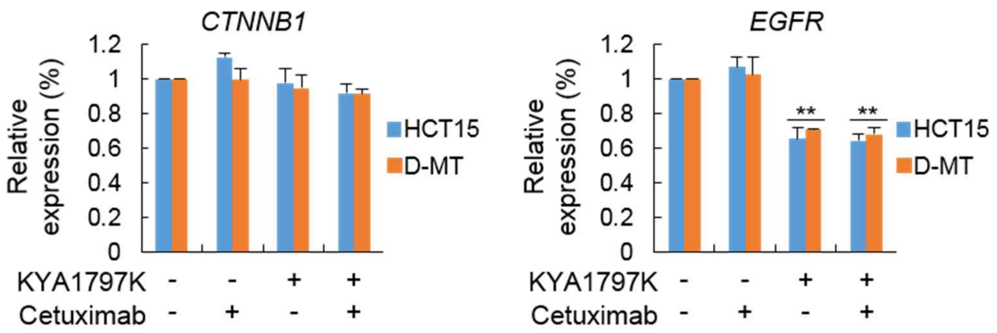

Supplementary Figure S5 The level of *EGFR* mRNA is reduced by KYA1797K. Real-time qPCR was performed to determine the mRNA levels of *CTNNB1* and *EGFR* in HCT15 or D-MT cells treated with the indicated conditions for 24 hr. Data are represented as mean ± SD (n = 3). Two-sided student's t test, $^{**}P < 0.005$.

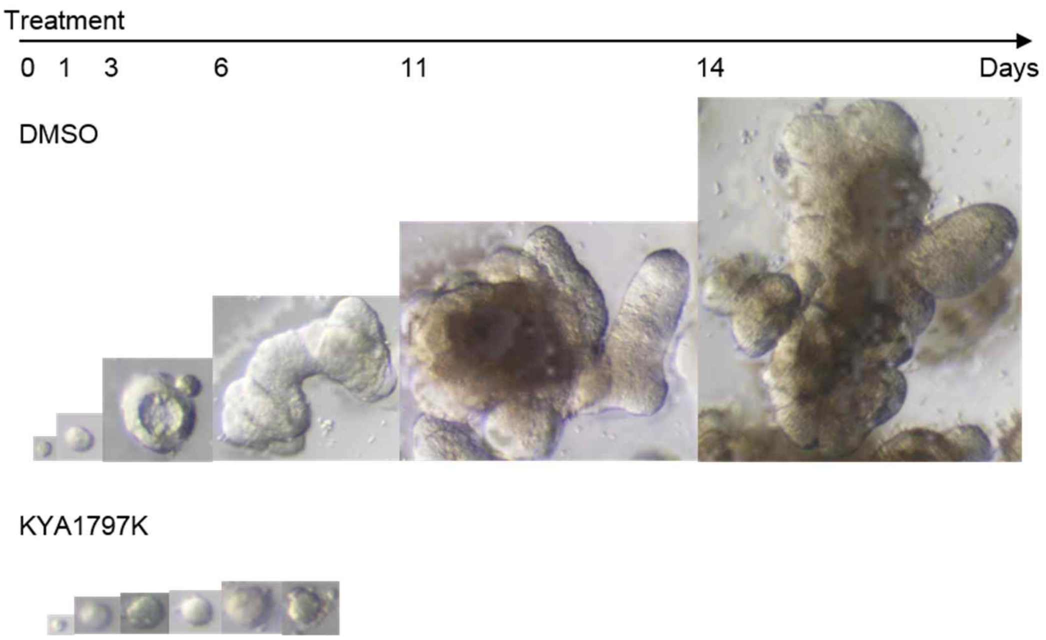

Supplementary Figure S6 The effectiveness of KYA1797K on formation of tumor organoids derived from a CRC patient tissue. The human tumor organoids were cultured and treated with DMSO or 20 μM of KYA1797K for 14 days. The representative serial DIC magnified images of tumor organoids were visualized at indicated time point (Days 0, 1, 3, 6, 11, and 14).

Supplementary Table S1. Real time-q PCR primer

GENE	Forward Primer	Reverse Primer
EGFR	ATGCCCGCATTAGCTCTTAG	GCAACTTCCCAAAATGTGCC
CCND1	GACCATCCCCCTGACGGCCGAG	CGCACGTCGGTGGGTGTGC
CTNNB1	ACAAGCCACAAGATTACAAGAA	GCACCAATATCAAGTCC AAGA
β-actin	AATCTGGCACCACACCTTCTAC	ATAG CACAGCCTGGATAGCAAC
